# 

**Published:** 1901-05

**Authors:** 


***NOTICE.—This JOURNAL and THE INTERNATIONAL JOURNAL OF SURGERY one year for $1.00 cash, to new subscribers. No out-of-town checks accepted unless 10c. is added for cost of cashing. Strictly to new subscribers.***

---

**This Journal and Atlanta Semi-weekly Journal one year for $1.0 cash in advance. No checks. Strictly to new subscribers.**

***NOTICE.—This JOURNAL and THE INTERNATIONAL JOURNAL OF SURGERY one year for $1.00 cash, to new subscribers. No out-of-town checks accepted unless 10c. is added for cost of cashing. Strictly to new subscribers.***

**This Journal and Atlanta Semi-weekly Journal one year for $1.00 cash in advance. No checks. Strictly to new subscribers.**

